# Acknowledgement to Reviewers of *Biosensors* in 2018

**DOI:** 10.3390/bios9010011

**Published:** 2019-01-10

**Authors:** Biosensors Editorial Office

**Affiliations:** MDPI, St. Alban-Anlage 66, 4052 Basel, Switzerland

Rigorous peer-review is the corner-stone of high-quality academic publishing. The editorial team greatly appreciates the reviewers who contributed their knowledge and expertise to the journal’s editorial process over the past 12 months. In 2018, a total of 131 papers were published in the journal, with a median time to first decision of 15 days and a median time to publication of 41 days. The editors would like to express their sincere gratitude to the following reviewers for their cooperation and dedication in 2018:
Abbas, BarfidokhtLiangdong, ZhuAbdul Rahim, FerhanLisa M, HarrisonAdam, MelvinLourdes Josefina Rivas, TorcatesAdrian, NeaguLu, XuAdrian, RuffLuca, LanotteAffar, KarimullahLucas, FerreiraAgnieszka, KrólickaLuigi, La SpadaAgostino, RomeoLuis, AbegãoAhyeon, KohLuis, CabralesAida, EbrahimiLuisa, GiansantiAkshay, KaleMadhu, SheriAlastair W., WarkMagdalena, OćwiejaAlberto, SinibaldiMałgorzata, PrzybytAlejandro, BaldominosMana, TomaAlessandro, ChiadòManuel, Aleixandre HerreroAlessandro, TonacciMarcin, GnybaAlessandro, PezzellaMarco, GrossiAlessandro, OrtisMarco, BuscagliaAlexander, KachynskiMaria, LermAlexandru, MüllerMaria, Soler AznarAlexey V., KrasnoslobodtsevMaria, PedreroAlfonso, Rodriguez-HerreraMaria, MassaoutiAlfredo, SánchezMaria, Gamella CarballoAlina, KarabchevskyMaria, João RamalhosaAlois, BonifacioMaría Carmen, Blanco-LópezAlphonsus, NgMaria Del Mar Ortega-villaizan, RomoAlphus, WilsonMaría Encarnación, Lorenzo AbadAmirhossein, ShahshahaniMaria Laura, ErminiAmitava, MoulickMariana, Medina-SánchezAmy M., WenMariana, IonitaAna, C.A. RoqueMariana Emilia, GhicaAna, LedoMaria-Nefeli, TsaloglouAna Dinora, Guzman-ChavezMario, RothbauerAna Isabel, BarbosaMario, CalveteAnastasios, EconomouMarketa, VaculovičováAndrea, GerbinoMarten, PostmaAndrea, MarcantoniMartina, ViefhuesAndreas, LeschMartina, ZangheriAndrew, ShoffstallMasaki, UjiharaAnitha, DevadossMatteo, GrattieriAnja Hviid, SimonsenMatti, HuotariAnoop, MenacheryMauro, EpifaniAntonio, BarberisMelani, SolomonAraceli, González CortésMelikhan, TanyeriArnaud, BuhotMeng, SunAron, HakonenMichael, SchöningArtem M., PlissMichael, KangasArturo, Martínez-RodrigoMichael, NortonArunas, RamanaviciusMichael, LangAsta, TamulevičienėMichele, SchiavonBal Ram, AdhikariMichihiko, NakanoBenoît, PiroMiguel, MorenoBernhard, RothMimma, NardelliBerta, Esteban-Fernandez De AvilaMin, ParkBishnu, RegmiMonica, FedeleBo, TianMoritoki, EgiBoon Giin, LeeMorteza, ArameshBoris B., DzantievMu-chun, WangBrendan D., MacDonaldMuhammad, KhanBryan, GannonMurilo, SanthiagoBurcu, GumuscuNaresh, KumarCarlo, CamerlingoNasrin, HooshmandCarlo, MassaroniNathalie, BissonnetteCarlos A., Ramírez-VargasNeftali, NuñezCarlos Andres, Galan-VidalNetzahualcóyotl, ArroyoCarlos Eric, Galvan-TejadaNicole, Jaffrezic-RenaultCatarina R. F., CaneiraNikhil, BhallaCees, OttoNirmal, GoswamiCélia, SilveiraNirmalya, TripathyCelia F., RodriguesNoemí, De Los Santos ÁlvarezChia-Chen, ChangNoureen, SirajChiara, FabrisOluwasesan, AdegokeChii-Wann, LinOnur, CakmakChiu-Yen, WangØrjan G., MartinsenChristian, RenkenPablo Ivan, NikelChristian, MayerPandiaraj, ManickamChristoph, SloukaPanneerselvam, RajapandiyanChristopher, LemonPaolo, BollellaChulhun, KangPaolo, BertoncelloConrad, RizalPartha, RayConstantin Mihai, LucaciuPasquale, RussoCristiana, CalicetiPatricia, KhashayarCristina, ComanPatricia, BroderickCristina, AriñoPatricia, Losada-PerezDae-Yoon, KimPau, HerreroDamion, CorriganPaul, MillnerDana, GrecovPaul Richard, BarberDaniel, Martín-YergaPaul W., BuehlerDanny K. Y., WongPawan, JollyDevon, FitzgeraldPeng, XuDietmar, KnoppPengtao, WangDiming, ZhangPetar, KassalDimitrios P., NikolelisPeter, JacobsDomenico, GrimaldiPeter, OwensDong, SeongPierangelo, BellioDong June, AhnPo, WangEhsan, SamieiPranay, AgarwalElias K., SpanakisPratik, ShahElizabeth, MannProsper, KanyongEmanuele Lindo, SeccoQiao, YangEn-Chih, ChangQingxiang, WangEnrico, CaianiRajashekhar, GangarajuEnrico, VeronaRamaraja P., RamasamyEric A.E., GarberRaquel, BailónEric De Souza, GilReinhard, MöllerErol, OzgurReinhold, OrglmeisterEstefanía, Costa RamaRejane L., PetersenEttore, MasseraRobert, HorvathEulalia, PereiraRoss, D. MiltonFan, ZhengRossano, LattanzioFederico, TascaRoza, SzwedaFeng, LiRui, LimaFengchun, TianRupesh K., MishraFiliz, YesilkoySabine, MüllerFiona, JamesSai, WangFirman Mangasa, SimanjuntakSaimon Moraes, SilvaFlavio Della, PelleSalvatore Gianluca, LeonardiFlorentina-Daniela, MunteanuSalzitsa, AnastasovaFlorina, TeodorescuSam H., AuFrancesca, CostantiniSandeep K., VashistFrancesco, RundoSankarasekaran, ShanmugarajuFrancisco Javier, Gimeno BlanesSara, Abalde-CelaFrank, DavisSara, TombelliFranz L., DickertSara Isabel Augusto, FateixaFrieder, SchellerSatish Balasaheb, NimseGabe A., KwongScott, WalperGabriel, CaffarenaSebastian, GutschGanesh, NarayananSebastian Andres, DiazGanesh Kumar, ManiSeok Jae, LeeGang, WeiSeong Soo A., AnGaspar, RegoSergey, ShityakovGeorg Christoph, BrunauerShailabh, KumarGiovanna, MarrazzaShilpa, SivashankarGiovanni, VozziShinji, KajimotoGiuseppe, MaruccioShuwen, ZengGoldie, OzaSimone, Del FaveroGregory W., BishopSimone, MorosiGreta, FaccioSnober, AhmedGuillaume, HaïatSonal, PadalkarGuodong, SuiSorah, YoonHai Jun, RongStefania, MarzoratiHalonen, NiinaStefano, CintiHaoyuan, ChenStefano, CiciliotHeidi, CulverStefano, LaiHenrik, PedersenStéphane, MarinescoHeongkyu, JuStewart, SmithHideyuki, MitomoSugawa, KosukeHirofumi, TaniSusan, Buckhout-WhiteHiroyuki, FurutaSusana, De Marcos RuizHugo, Posada-QuinteroSven T., StrippHui, FangTaewoo, KimHung-Yu, ChangTakeo, MinamikawaHutomo S., WasistoTamás, PardyHyeon-Yeol, ChoTania, GarcíaHyungwoo, KimTao, YueIbrahim, AbdulhalimTatiana, ZamayIcíar, GonzálezTeruhiko, MatsubaraIgor, ZozoulenkoThomas, DoneuxIliana, FauziTim, PrickettIngemar, FredrikssonToshikatsu, TakanamiItthipon, JeerapanTrieu, NguyenJ.-Pablo, SalvadorTuo, JiJacek, WojtasTzu-En, LinJacek, NamieśnikUlrich, HofmannJaemin, KimUlrike, KluehJae-Sung, KwonUwe, SchnakenbergJaewook, LeeVaidotas, MarozasJagotamoy, DasValentina, GrippoJames B., KotconVangelis, EconomouJan, NedomaVenkata D. B. C., DasireddyJanis, SpigulisVenkatesh, A GovindarajJasmina, VidicVerónica, Montes-GarcíaJayakrupakar, NallalaViacheslav, NikolaevJeon Woong, KangVictor Constantin, DiculescuJesús, Lozano RogadoVijayalakshmi, VelusamyJiajia, XueVitali, SikirzhytskiJianfeng, WuVladimir, SukhovJianfeng, PingVladimir A., BaulinJing, LiuVladislav, KhrustalevJinnan, ZhangVlastimil, VyskočilJinxing, LiangWan-Young, ChungJinyoung, LeeWei Suong, TEOJochen, LangWuliang, YinJohn, MitchellXiangcheng, SunJonathan T., ReederXiao, LiJong I, RheeXiao Dong, ZhouJong-Hoon, KimXosé Antón Vila, SobrinoJoo Chuan, YeoXue, QiuJoonseok, LeeXuefei, GaoJörg, FitterXuewei, WangJorge, HenriquesYang Jun, KangJosé, Juan-ColásYang-chun, YongJosé Machado, Da SilvaYannick, SaalbergJosé Manuel, ParedesYasuhisa, OmuraJuan Antonio, Corrales RamonYasuko, ObataJuan Carlos, Martinez EspinosaYi, FangJuan Francisco, ArmijoYiyan, FeiJun, SuzurikawaYiyan, LiJun, ZhangYiyao, Ye-LinJun, AndoYohei, JinnoJun-ichi, AnzaiYongki, ChoiKamrul, IslamYongxin, ZhaoKarol, MalechaYu-Chun, KungKatrin, PollmannYue, ZhuoKazuhiro, AokiYuen, ClementKen-ichi, NomuraYuksel, TemizKien Voon, KongYun, WangKosuke, InoYunlei, XianyuKuangwen, HsiehYunze, YangKyle Elliott, MathewsonYuri E., NesmelovKyriaki, ManoliZahra, TaleatLaena, PernomianZhe, GaoLaura, MicheliZhigang, ZhuLaura, HeitmanZhiguo, ZhangLaurent, DavidZhiqiang, Cheng

